# Inherent P2X7 Receptors Regulate Macrophage Functions during Inflammatory Diseases

**DOI:** 10.3390/ijms23010232

**Published:** 2021-12-26

**Authors:** Wenjing Ren, Patrizia Rubini, Yong Tang, Tobias Engel, Peter Illes

**Affiliations:** 1 International Collaborative Centre on Big Science Plan for Purinergic Signalling, Chengdu University of TCM, Chengdu 610075, China; pathlesswoods@126.com (W.R.); patrizia.rubini@gmx.de (P.R.); tangyong@cdutcm.edu.cn (Y.T.); 2 School of Acupunct3ure and Tuina, Chengdu University of TCM, Chengdu 610075, China; 3 Department of Physiology and Medical Physics, Royal College of Surgeons in Ireland, University of Medicine and Health Sciences, D02 YN77 Dublin, Ireland; tengel@rcsi.ie; 4 FutureNeuro, SFI Research Centre for Chronic and Rare Neurological Diseases, RCSI University of Medicine and Health Sciences, D02 YN77 Dublin, Ireland; 5 Rudolf Boehm Institute for Pharmacology and Toxicology, University of Leipzig, 04107 Leipzig, Germany

**Keywords:** macrophages, P2X7R, pore formation, inflammasome activation, inflammatory diseases

## Abstract

Macrophages are mononuclear phagocytes which derive either from blood-borne monocytes or reside as resident macrophages in peripheral (Kupffer cells of the liver, marginal zone macrophages of the spleen, alveolar macrophages of the lung) and central tissue (microglia). They occur as M1 (pro-inflammatory; classic) or M2 (anti-inflammatory; alternatively activated) phenotypes. Macrophages possess P2X7 receptors (Rs) which respond to high concentrations of extracellular ATP under pathological conditions by allowing the non-selective fluxes of cations (Na^+^, Ca^2+^, K^+^). Activation of P2X7Rs by still higher concentrations of ATP, especially after repetitive agonist application, leads to the opening of membrane pores permeable to ~900 Da molecules. For this effect an interaction of the P2X7R with a range of other membrane channels (e.g., P2X4R, transient receptor potential A1 [TRPA1], pannexin-1 hemichannel, ANO6 chloride channel) is required. Macrophage-localized P2X7Rs have to be co-activated with the lipopolysaccharide-sensitive toll-like receptor 4 (TLR4) in order to induce the formation of the inflammasome 3 (NLRP3), which then activates the pro-interleukin-1β (pro-IL-1β)-degrading caspase-1 to lead to IL-1β release. Moreover, inflammatory diseases (e.g., rheumatoid arthritis, Crohn’s disease, sepsis, etc.) are generated downstream of the P2X7R-induced upregulation of intracellular second messengers (e.g., phospholipase A2, p38 mitogen-activated kinase, and rho G proteins). In conclusion, P2X7Rs at macrophages appear to be important targets to preserve immune homeostasis with possible therapeutic consequences.

## 1. Introduction

**Figure 1:**
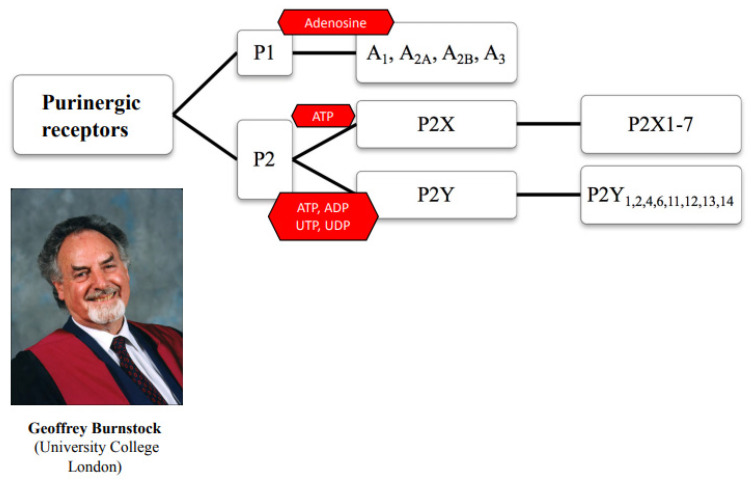
Classification of purinergic receptors. P1Rs consist of four subtypes and respond to the endogenous agonist adenosine. P2Rs consist of two subtypes, the ligand-gated cationic channels P2X (P2X1–7) responding to ATP only, and the G protein-coupled receptors P2Y (P2Y_1,2,4,6,11,12,13,14_). The principal agonists of P2YRs are the following: ATP/ADP (P2Y_1,11,12,13_), UTP/UDP (P2Y_2,4,6_), and UDP-glucose (P2Y_14_). Purinergic receptors were classified by the late Geoffrey Burnstock (see photo), together with his colleague Maria Pia Abbracchio [3].

While millimolar concentrations of ATP are stored in the cell interior, where they are used under anaerobic conditions as an energy supply, this nucleotide can also escape to the extracellular space through discontinuities generated by metabolic/mechanical damage to the cell membrane or by means of membrane transporters and channels. Extracellular ATP has been characterized as a signaling molecule coordinating cellular and, thereby, whole organism functions [1,2]. Extracellular ATP or its enzymatic breakdown products, ADP, AMP and adenosine (see below), may then stimulate a range of membrane receptors (Rs) [3]. These receptors are classified as belonging to two types termed P2 and P1 (Figure 1). In addition, P2Rs can be subdivided into the ligand-activated P2X [2,4,5], and the G protein-coupled P2Y receptor types [6,7]. Adenosine acts on the P1 receptor type, which is also G protein-coupled. P1Rs are either stimulating (A2A, A2B) or inhibiting (A1, A3) adenylate cyclase production via the mediation of G proteins [8]. A further classification identifies seven mammalian subtypes of P2XRs (P2X1-7) and eight mammalian subtypes of P2YRs (P2Y_1_, P2Y_2_, P2Y_4_, P2Y_6_, P2Y_11_, P2Y_12_, P2Y_13_, P2Y_14_). Whereas P2XRs respond only to ATP, P2YRs respond to ATP/ADP, UTP/UDP, or UDP-glucose. Signaling through P2/P1Rs is rapidly terminated by the conversion of ATP to adenosine and eventually to the inactive inosine within the extracellular space by the activity of ecto-nucleotidases [9,10]. The four major groups of ecto-nucleotidases are the ecto-nucleoside triphosphate diphosphohydrolases (NPTDases), ecto-5’-nucleotidase, ectonucleotide pyrophosphatase/phosphodiesterases, and alkaline phosphatases. Three related family members of NPTDase are expressed in the mammalian brain [11]. NTPDase1 (CD39) hydrolyses nucleoside-5’-triphosphates and -diphosphates eventually to nucleoside-5’-monophosphates equally well. Ecto-5’nucleotidase (CD73) degrades nucleoside-5’monophosphates to the respective nucleoside, e.g., AMP to adenosine.

ATP binds to P2X and, after degradation into its metabolites ADP and adenosine, indirectly activates P2Y and P1Rs, respectively; P2/P1Rs are located at virtually all subsets of immune cells, which has been recognized to be related to a range of biological actions in the immune system [12]. P2X7Rs are distinguished from other P2XRs by their longer C-termini and a very low affinity for ATP when compared to the other P2XRs; this is thought to be the cause of their participation in pathophysiological reactions [13,14]. The P2X7R is widely distributed and functional at the innate cells of the adaptive immune system constituting the first line of defense against invading pathogens. These cells are lymphocytes, granulocytes, macrophages, dendritic cells in peripheral tissues [15,16], and microglia, the resident macrophages of the central nervous system (CNS) [17]. An increasing number of studies show that P2X7Rs play a crucial role in the functions of macrophages by stimulating: (1) the inflammasome, leading to the production of interleukin-1β (IL-1β) and IL-18; (2) the stress-related protein kinase pathway, resulting in apoptosis; (3) the mitogen-activated protein kinase pathway, leading to generation of reactive oxygen and nitrogen species; and (4) phospholipase D, initiating phagosome/lysosome fusion [18]. This review focuses on the state of our present knowledge about P2X7Rs in macrophages during inflammatory processes, and discusses the possibility of P2X7Rs as therapeutic targets to alleviate the deleterious consequences of macrophage activation.

## 2. The P2X7R at Macrophages

Macrophages were first defined by Elie Metchnikoff (1845–1916) and characterized by their phagocytotic activities to maintain tissue repair and integrity [19]. They are unique innate immune cells that play a prominent role in the host defense by virtue of their ability to rapidly recognize, engulf, and kill pathogens and apoptotic cells critically required for the maintenance of homeostasis. Macrophages are remarkable mononuclear phagocytes that efficiently clear approximately 2 × 10^11^ erythrocytes each day and remove the worn-out cells and debris generated by tissue remodeling [20]. Macrophages are derived from monocytic precursors in the blood and bone marrow. However, growing evidence suggests that tissue-resident, or tissue-specific, macrophages are embryonically derived and self-renewing [21]. The tissue-resident macrophages are also generated from yolk sac progenitors in spleen, liver, lung, skin, and brain [22]. These macrophages are persistent and are maintained into adulthood by embryonic progenitors, without being replaced by bone marrow- and blood monocyte-derived cells [23]. Macrophages are distributed to various organs and are spread over the whole body, including Langerhans cells of the skin, Kupffer cells of the liver, marginal zone macrophages of the spleen, alveolar macrophages of the lung, and microglia of the CNS [20,24].

The ability of the immune system to recognize molecules that are broadly shared by pathogens is, in part, due to the presence of immune receptors called toll-like receptors (TLRs). In response to TLR ligands (e.g., lipopolysaccharide [LPS], a constituent of the cell membrane of gram negative bacteria) and the cytokine interferon-γ (IFN-γ), macrophages undergo activation to a pro-inflammatory, classic type termed M1 [25,26]. M1 macrophages are amoeboid in shape, are able to phagocytose pathogenic bacteria, and typically release destructive inflammatory mediators such as pro-inflammatory cytokines (interleukin-1β [IL-1β], tumor necrosis factor-α [TNF-α]), chemokines, proteases, reactive oxygen/nitrogen species, and probably also the excitotoxic ATP and glutamate by vesicular exocytosis [27,28]. Macrophages may also undergo M2 activation, which clear cellular debris through phagocytosis and release numerous protective factors (IL-4, IL-13, nerve growth factor [NGF], fibroblast growth factor [FGF]) [25,26]. M2 macrophages, which can be further divided into M2a, M2b, M2c, and M2d, are characterized by promotion of tissue remodeling, and tumor progression that are associated with resolution of chronic inflammation [25,29,30]. These highly specialized cells often contribute to tissue homeostasis in all organs.

**Figure 2:**
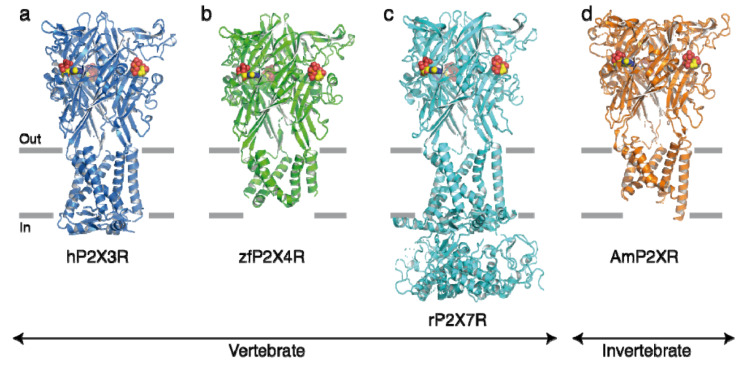
Structures of selected P2XRs. (**a**) Structure of the human P2X3R bound to ATP [34]. The hP2X3R is shown in blue; ATP is shown as spheres in a–d (carbon is yellow, oxygen is red, nitrogen is blue, and phosphorus is orange). Horizontal grey bars indicate the approximate location of the membrane bilayer defining the extracellular (out) and intracellular (in) milieu. (**b**) Structure of the zebrafish P2X4R bound to ATP [35]. The zf P2X4R is shown as a green. (**c**) Structure of the rat P2X7R bound to ATP [36]. The rP2X7R is shown in cyan. (**d**) Structure of the invertebrate *Amblyomma maculatum* P2XR receptor bound to ATP [37]. The AmP2XR is shown in orange. Note the structural similarity between vertebrate and invertebrate P2XRs. For structures having undergone heavy truncations, some membrane spanning helices, as well as the N- and C-termini are lacking in their intracellular sides. Reproduced from [31] with permission.

P2XRs show similar tertiary and quaternary architecture, further confirming the hypothesis that all P2XRs belong to the same structural and evolutionary group (Figure 2) [31]. The P2X7R is a non-selective cationic channel gated by high concentrations of ATP leading to permeation of Na^+^, K^+^, and Ca^2+^ [13,14]. While already somewhat lower concentrations of ATP open the cationic channel, still higher concentrations, especially on repeated application, will create a much larger aqueous pore and allows permeation to molecules of up to 900 Da [13,32] (see later). The P2X7R, similar to the other members of the P2XR family, consists of three subunits (one large extracellular loop, two transmembrane regions, and N- and C-terminal ends) forming a receptor, but each subunit has a much longer C-terminus than that of the other P2XRs [14,33]. The agonist-binding pouch is located at the contact points of two neighboring subunits. There is an increasing recognition that P2X7Rs plays a pivotal role in virtually all immune cell types, especially macrophages [12].

The P2X7R is widely expressed by myeloid and lymphoid immune cells, as well as by mast cells [15,31]. P2X7Rs can be detected in the majority of blood monocytes, and the expression of immunoreactive P2X7Rs is greatly enhanced, almost 10-fold, as these monocytes develop into differentiated macrophages [38]. Thus, the expression of P2X7Rs is augmented after inflammation, triggering monocytes to differentiate into macrophages [32,39]. The expression of P2X7Rs on monocytes/macrophages was four- to five-fold greater than on lymphocytes because of the larger size and surface area of the former cell type. In contrast, P2X7Rs have been shown to be weakly expressed on neutrophils and platelets [40]. The increased expression of P2X7Rs in activated macrophages was associated with the ability of these cells to differentiate into multinucleated giant cells to form syncytia [39]. The proliferation, differentiation, and apoptosis of macrophages were also mediated by P2X7R activation [41,42]. Recently, it became increasingly apparent that P2X7Rs play a potential role in the macrophage immune responses to pathogens and disorders, such as inflammation [43], cancer [44], oxidative stress [45], and virus infection [46].

## 3. The Regulation of Pore Formation on Macrophages via P2X7Rs

The P2X7R is abundantly expressed on macrophages and exhibits a variety of functions in innate and adaptive immune responses. Numerous studies reveal that the sequelae of P2X7R activation on macrophages includes the generation of membrane currents [47,48], membrane permeabilization, uptake of large molecules [49], massive perturbations of Na^+^, K^+^, and Ca^2+^ homeostasis [24,50], inflammasome activation, and interleukin processing [51,52,53]. Cell membrane blebbing [47,54] and spontaneous cell fusion [55] of macrophages are also mediated by P2X7Rs.

As already mentioned, the activation of P2X7Rs has an ability to open a typical ion channel for small cations, including Ca^2+^, and subsequently a particular membrane pore for larger molecules (e.g., the fluorescent dyes YO-PRO-1 and ethidium bromide). It has been shown that intracellular Ca^2+^ in murine peritoneal macrophages [52] or human macrophages [47] is elevated after the activation of P2X7Rs by a high concentration of ATP. Although a current carried by the inward flux of Na^+^ and Ca^2+^ is generated after the stimulation of all P2XRs (including P2X7Rs), the degree of current desensitization is quite diverse [56]. Noteworthy, P2X7Rs are considered slow desensitizers and, therefore, their activation leads to a continuous, long-lasting influx of Ca^2+^.

Repetitive activation of P2X7Rs with ATP evoked a biphasic membrane current in human macrophages, which is supposed to reflect large plasma membrane pore generation for the polyatomic cationic dye YO-PRO-1 [47]. Upon stimulation with ATP, a whole-cell membrane current is activated immediately that initially desensitizes and subsequently facilitates upon prolonged channel opening; P2X7Rs promote cation-selective dye uptake while excluding anions (such as anionic calcein) [47]. The application of P2X7R antagonists, such as A-438079 and A-804598, prevents both the current responses to ATP and the ATP-induced uptake of large cationic fluorescent dyes in cultured human microglia [57]. This process is related to several other immunological functions, especially in inflammasome activation [58,59]. Some other effects, such as activation of p38 MAP kinase [60,61], activation of phospholipase D [62,63], the production of reactive oxygen/nitrogen species [56], and killing of *Mycobacterium tuberculosis* [64,65] were also involved in the opening of large membrane pores. P2X7R occupation was further associated with the release of IL-1β, interferon-γ, and reactive oxygen/nitrogen species in murine macrophages. Pore formation is essential for triggering ATP-induced interleukin processing in the immune system.

A particularly intensively discussed issue is whether the initially opened cationic channel dilates and, thereby, establishes the larger diameter pore permeable to cationic dyes or whether two different channels are involved in this effect [66]. Originally, it was suggested, based on equilibrium potential (V_rev_) measurements with the whole-cell patch clamp technique, that the ion conducting pathway undergoes progressive dilation [67]. However, this suggestion was recently refuted, because the shift in V_rev_ in a medium in which the counter ion of intracellular K^+^ was NMDG^+^ instead of Na^+^, emerged due to time-dependent alterations in the concentration of intracellular ions rather than channel dilation [68]. Moreover, during long-lasting activation of P2X7Rs, the single-channel current amplitude and the permeation characteristics remained constant [69].

The P2X7R C-terminal tail constitutes about 40% of the whole protein; it distinguishes P2X7Rs from the other P2XR types and plays a key role in P2X7R ion channel function [70]. The deletion or massive truncation of the C-terminus prevents effects mediated by receptor activation, such as dye uptake and membrane blebbing, but also alters channel kinetics [71]. In addition, there is indication that P2X7R-induced cytolytic pore formation at macrophages is regulated by its unique C-terminal domain [72].

**Figure 3:**
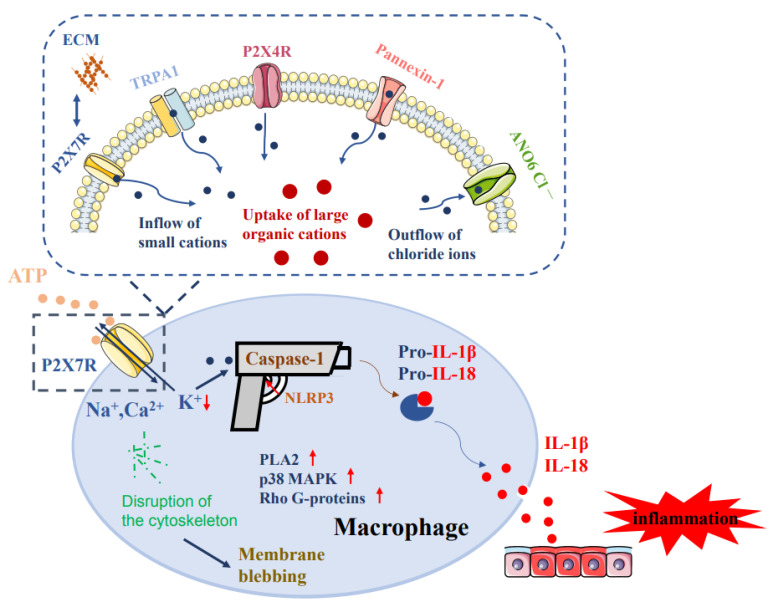
Role of P2X7Rs in macrophages in inducing inflammation. The activation of P2X7Rs by high concentrations of ATP drives the influx of Na^+^/Ca^2+^ and efflux of K^+^ through this plasma membrane-localized receptor channel. A decrease in the intracellular K^+^ concentration is, in co-operation with the stimulation of the toll-like receptor 4 (TLR4) by lipopolysaccharide, a stimulus for the composition and activation of the nucleotide-binding, leucine-rich repeat, pyrin domain containing 3 (NLRP3). NLRP3 then activates caspase-1, which degrades pro-interleukin-1β (pro-IL-1β) to IL-1β. IL-1β is released from macrophages either by membrane diffusion or packed in extracellular vesicles. P2X7R stimulation also leads to the activation of phospholipase A2 (PLA2), p38 mitogen-activated kinase (MAPK), and rho family G-proteins. The pro-inflammatory cytokines IL-1β and IL-18 cause inflammation. In addition, the stimulation of P2X7Rs initiate the opening of large membrane pores in co-operation with the extracellular matrix (ECM), transient receptor potential A1 (TRPA1) channels, P2X4R-channels, pannexin-1 hemichannels, and anoctamin (ANO6) Cl^-^ channels. These membrane pores allow the uptake of otherwise non-permeable cationic molecules of up to 900 Da, triggering the already-mentioned further effects of the P2X7R. Figuratively spoken, P2X7Rs provide bullets to NLRP3/caspase 1, and NLRP3 triggers the gun to release the pro-inflammatory cytokines (see the central part of the lower panel).

Pannexins are a family of vertebrate proteins identified by their homology to the invertebrate innexins [73]. While innexins are responsible for forming gap junctions in invertebrates, the pannexins have been shown to predominantly exist as large transmembrane channels connecting the intracellular and extracellular space, allowing the passage of ions and small molecules between these two compartments. In contrast to pannexins, connexins are gap junction forming proteins in mammals which may also exist in the hemichannel form exerting analogous effects to pannexins. Pannexin-1 has been identified as a P2X7R-associated protein [74,75]; it is an ATP-permeable channel ubiquitously expressed in macrophages and activation of the pannexin-1 channel mediates ATP release to induce macrophage cell death [76] (Figure 3). This channel was suggested to be responsible for the activation of the large pore induced by P2X7Rs in macrophages and was identified as an upstream molecule essential for the activation of the inflammasome/caspase-1/IL-1β complex in macrophages [59]. The same authors reported that pannexin-1 co-immunoprecipitates with the P2X7R protein, and selective inhibition of pannexin-1 reverses P2X7R-mediated dye uptake without altering P2X7R protein expression [59]. Thus, pannexin-1 in the macrophage membrane has been identified to be a large pore stimulated by P2X7Rs.

However, pannexin-1-induced dye uptake is also observed independent of P2X7R occupation by ATP [77]. Moreover, it was also reported (in contrast to the previously mentioned data [74]) that the pannexin-1 antagonist probenecid and interference RNA (RNAi) targeting of pannexin-1 did not affect the P2X7R macroscopic current in mouse peritoneal macrophages [75]. Similarly, it was found that a range of pannexin-1 inhibitors did not block the ATP-induced cationic dye uptake in cultured human microglia [57].

However, the activation of Cl^−^ channels appears to be necessary for ATP-induced dye uptake in mouse and human macrophages, and the activity of caspase-1, which is essential for interleukin release, is also blocked by Cl^−^ channel antagonists [57]. Anoctamin 6 (ANO6), a putative Ca^2+^-activated Cl^−^ and non-selective anionic channel, contributes to P2X7R pore formation, and YO-PRO-1 uptake produced by P2X7R activation is attenuated by knockdown of ANO6 [54]. ANO6 may, therefore, constitute another downstream target of P2X7Rs in macrophages. Moreover, P2X7Rs interact with P2X4Rs via their C-termini and disruption of the P2X7/P2X4R interaction hinders responses to high concentrations of ATP in murine macrophages [78].

These rather conflicting results regarding the P2X7R pore formation suggest that more than one channel is probably responsible for dye uptake, and pannexin-1 and/or ANO6 associated with P2X7Rs participate to a certain extent in this process. Nevertheless, a possibility that pannexin-1 allows dye uptake by altering the function of plasma membrane lipids and membrane transporters cannot be ruled out either. In fact, the panda P2X7R, when purified and reconstituted into liposomes, forms a dye permeable pore in the absence of other cellular components [79]. P2X7R channel activity is facilitated by phosphatidylglycerol and sphingomyelin, but dominantly inhibited by cholesterol. Thus, the P2X7R constitutes a lipid composition-dependent dye-permeable pore, whose opening is facilitated by palmitoylated cysteines near the pore-lining helix.

Along this line of thinking, it was recently suggested that current facilitation and macropore formation involve functional complexes comprised of P2X7R and TMEM16, a family of Ca^2+^-activated ion channel/scramblases [80]. Macropore formation entails two distinct large molecular permeation components, one of which requires functional complexes featuring the TMEM16F subtype, the other likely being direct permeation through the P2X7R pore itself. This idea perfectly complements the previous view that the P2X7R channel allows the passage of large cationic molecules immediately from its initial activation, but at a much slower pace than that of the small cations Na^+^, K^+^, and Ca^2+^ [16,81].

Membrane blebbing is a characteristic feature of injured cells [82]. Extensive membrane blebbing in murine and human macrophages occurs after prolonged activation of P2X7Rs [54,55,83]. This process is completely blocked by the P2X7R antagonist A-804598 [47], and is associated with reversible disruption of the cytoskeleton, activation of phospholipase A2 (PLA2), p38 MAPK, and Rho G-proteins [47,60,84]. P2X7R driven K^+^ efflux and Ca^2+^ influx is also involved in macrophage membrane blebbing.

The macrophage exocytosis, proliferation and cell morphology are regulated by P2X7Rs as well. Convincing evidence proves that P2X7Rs play a crucial role in macrophage exocytosis induced by single-walled carbon nanotubes [85]. The proliferation of microglia almost completely depends on P2X7Rs, and microglia density is also decreased in P2X7R deficient embryos [86]. Additionally, IL-1β, mediated by activation of the P2X7R pore, is crucial for microglial proliferation [87]. Ionized calcium binding adaptor molecule 1(Iba1), as a cytoskeleton protein specific only for microglia and macrophages, is frequently involved in cell migration, and Iba1 silencing enhances P2X7R function [88].

## 4. The Role of P2X7Rs at Macrophages in Inflammatory Diseases

The P2X7R pore is believed to be an essential component of regulating inflammasome activation in macrophages [33,89]. Inflammation leads to cell damage and massive outflow of ATP into the extracellular space. Macrophages are equipped with a battery of pattern recognition receptors that stereotypically detect pathogen-associated molecular patterns (PAMPs), such as LPS, from bacterial infection or danger-associated molecular patterns (DAMPS), such as ATP [66,90,91]. Activation of macrophages stimulates the release of IL-1β in a two-steps process: the first being the stimulation of toll-like receptor 4 (TLR4) by LPS, leading to accumulation of cytoplasmic pro-IL-1β, and the second being the ATP-dependent stimulation of P2X7Rs, promoting nucleotide-binding, leucine-rich repeat, pyrin domain containing 3 (NLRP3) inflammasome-mediated caspase-1 activation and secretion of IL-1β [92,93,94,95]. Caspase-1 generates IL-1β from pro-IL-1β by enzymatic degradation. It is important to note that the decrease of intracellular K^+^ after P2X7R stimulation initiates P2X7R-dependent NLRP3 inflammasome activation [15,96].

Indeed, brief stimulation of P2X7Rs is sufficient to initiate the processing of IL-1β in macrophages [97,98]. There is considerable evidence linking P2X7R function with caspase-1 and TLR4 signaling pathways, and prolonged P2X7R-ion channel stimulation is known to induce cytolytic cell death and Ca^2+^ overload as a macrophage death trigger [99]. The release of the typical pro-inflammatory cytokines IL-1β and IL-18 can be observed after about 20–30 min of P2X7R activation and leads to macrophage death [100].

P2X7R signaling also protects against bacterial infections through enhancing bacterial killing by macrophages, which is independent of the inflammasome. ATP release through connexin channels is instrumental in inhibiting inflammation and bacterial burden, and ATP protects against sepsis through activation of P2X7Rs in macrophages by enhancing intracellular bacterial killing [101].

In addition to the classic inflammatory pathways, the interaction between the P2X7R and other proteins located on macrophages is also essential for the inflammatory process. The P2X7R cross-talk with the extracellular matrix (ECM) can be exerted via paxillin. Paxillin is a multi-domain protein that localizes at the ECM; ATP triggers a P2X7R-paxillin interaction in murine and human macrophages to promote NLRP3 deubiquitination and K^+^ efflux [102].

Additional inflammasome-independent but P2X7R-dependent pro-inflammatory mechanisms have also been described. (1) A recent study suggested that transient receptor potential ankyrin 1 (TRPA1) was considered to co-localize with P2X7Rs in human cultured macrophages; it mediates Ca^2+^ influx in response to BzATP, partly inhibited by pharmacological blockers of TRPA1 [103]. This process is related to ATP-induced oxidative stress and inflammation. (2) P2X7Rs mediate the pro-inflammatory function of human beta-defensin 2 (HBD2) and human beta-defensin 3 (HBD3), although none of these molecules interact directly with P2X7R but rather induce the release of ATP [104]. (3) The stimulation of P2X7Rs by ATP enhances the transport of extracellular cGAMP into macrophages and subsequently activates STING, which is crucial for the anti-tumor immune response [44].

In contrast to their role in pro-inflammation, P2X7Rs might play an anti-inflammatory role in M2 macrophages [105]. The P2X7R function on anti-inflammation depends on the release of potent anti-inflammatory proteins, such as Annexin A1 [105]. Furthermore, in intermediate M1/M2-polarized macrophages, extracellular ATP acts through its pyrophosphate chains, to inhibit IL-1β release by other stimuli through two independent mechanisms: (1) inhibition of ROS production and (2) trapping of the inflammasome complex through intracellular clustering of actin filaments [106,107].

## 5. Peripheral Inflammatory Diseases

Multiple studies indicate that the activation of P2X7Rs in macrophages is involved in peripheral inflammatory diseases, including rheumatoid arthritis [53], Crohn’s disease [108], liver fibrosis [109,110], sepsis [101,111], renal inflammation [112,113], and pulmonary inflammation [114]. Almost all peripheral inflammatory diseases share the same, classic inflammatory pathway in macrophages with the following components: P2X7R stimulation, NLRP3 and caspase-1 activation, and IL-1β/IL-18 release. However, the fine tuning of the inflammatory pathways is different in various diseases and pathological states.

In rheumatoid arthritis patients, ATP release and P2X7R activation are significantly increased by anti-citrullinated protein antibodies (ACPAs) which are targeted against citrullinated proteins/peptides utilized as rheumatoid arthritis biomarkers [53]. ACPAs promoted IL-1β production by macrophages derived as peripheral blood mononuclear cells. ACPAs interacted with CD147 to enhance the interaction between CD147 and integrin β1 and, in turn, activated the Akt/NF-κB signaling pathway. The nuclear localization of p65 promoted the expression of NLRP3 and pro-IL-1β, resulting in priming.

The involvement of P2X7R-mediated NLRP3 inflammasome activation in IL-1β production in mouse macrophages supplemented with human hepatic stellate cells might contribute to extracellular matrix deposition and suggests that blockade of the P2X7R-NLRP3 inflammasome axis represents a potential therapeutic target for liver fibrosis [109,110]. P2X7Rs have been shown to modulate human THP-1 macrophages interacting with human hepatocytes in cell culture, thereby regulating lipid accumulation in hepatocytes [52].

However, P2X7Rs play a totally different role in sepsis. P2X7R activation on mice peritoneal macrophages suppresses sepsis-induced inflammation and augments killing of intracellular bacteria; connexin hemichannels may have a role in this process [101]. However, an opposite idea was also presented. In sepsis, a systemic blockade of P2X7Rs mitigates inflammatory responses and maintains intestinal barrier function partly by inhibiting the activation of M1 macrophages via the ERK/NF-κB pathway [111].

Cytokine storm, defined as macrophage activation syndrome (MAS), is characterized by the release of multiple pro-inflammatory cytokines (IL-1β, IL-18, IL-6, IL-2, IL-7, TNF-α) and chemokines (CCL2, CCL3) from macrophages [115]. MAS is involved in coronavirus disease-19 (COVID-19), and the blockade of pro-inflammatory cytokines by an anti-IL-6 or IL-6R antibody, such as Tocilizumab, has already been employed for the treatment of MAS and COVID-19 in patients [116,117]. The recombinant human IL-1 receptor antagonist Anakinra is also considered to be a possible therapy for COVID-19 in patients [118]. The numerous similarities between COVID-19 symptoms and those induced by P2X7R activation make it likely that low molecular weight P2X7R antagonists are probably appropriate therapeutic approaches for this disease [119].

## 6. Neuroinflammation

In the meantime, it is a well-known fact that P2X7Rs amplify CNS damage in neurodegenerative diseases, such as Alzheimer’s Disease, Parkinson’s disease, amyotrophic lateral sclerosis, multiple sclerosis, post-ischemic conditions, and neurodegeneration as a cause or consequence of epilepsy [120]. It is equally broadly accepted that the resident macrophages of the brain, the P2X7R-bearing microglial cells, mediate this effect. Extracellular β-amyloid (Aβ), a pathognomonic hallmark of Alzheimer’s disease, is surrounded by microglia, and the stimulation of P2X7Rs by high local concentrations of ATP originating from damaged CNS cells results in degeneration of nearby neurons [121]. Genetic depletion or pharmacological inhibition of P2X7Rs ameliorated the symptoms observed in various Alzheimer’s disease mouse models [122].

The expression of microglial P2X7Rs has been enhanced in a rat model of Parkinson’s disease, induced by the intranigral injection of LPS; the application of the P2X7R antagonist Brilliant Blue G (BBG) reduced the activation of microglia and the loss of nigral dopamine neurons by decreasing the phosphorylation level of p38 MAPK [123]. Under these conditions, the deleterious effects of microglial activation are due to the release of cytokines, nitric oxide, and reactive oxygen species [124].

P2X7Rs play a two-fold role in the course of amyotrophic lateral sclerosis (ALS) depending on the disease state [125]. BBG decreased microgliosis in an ALS mouse model at late pre-onset rather than at the asymptomatic or pre-symptomatic phases [125]. Consistent with the previous results, the application of the P2X7R antagonist A-804598 also alleviated disease progression in late pre-onset ALS by blocking the phagocytotic activity of SOD-1-G93A mouse microglia [126]. P2X7R antagonists modulate ALS progression by changing the polarization of microglia in SOD1-mutant mice [125,126]. At the late pre-onset phase of the disease, the overexpression of M1 microglial markers is downregulated by BBG treatment, while the anti-inflammatory M2 markers are concomitantly upregulated.

Similar changes in microglial polarization were observed at the later stage of multiple sclerosis or its rodent model, experimental autoimmune encephalomyelitis (EAP) [127]. In addition, P2X7Rs were reported to increase IL-1β, IL-6, and TNF-α release from microglia to induce neuroinflammation at a very early stage of multiple sclerosis, and the prophylactic use of P2X7R antagonists were found to delay the onset and to ameliorate disease progression in the mouse model of EAP [128].

P2X7R expression on microglia was increased during epilepsy induced by the intra-amygdala injection of kainic acid; P2X7R antagonists reduced the accompanying microgliosis and produced enduring suppression of epileptic seizures in mice injected with intra-accumbal kainic acid [129]. The typical P2X7R-dependent inflammatory pathways, including IL-1β, may participate in the ensuing neuronal damage [130].

Microglial P2X7Rs participate also in post-ischemic neurodegeneration. P2X7Rs are widely expressed on microglial cells in both hemispheres after a monolateral brain infarction; in this case, transition from the M1 to the M2 state occurs and a partial protective effect against the post-ischemic neurological impact develops in rats [131].

## 7. P2X7R Splice Variants and Polymorphisms

A number of P2X7R isoforms derived from alternative splicing were identified both in humans and rodents [31,132]. Some variants are expressed and functional, for example the human (h) P2X7B-R, and mouse and rat P2X7R variants “k”. In addition, several non-synonymous, intronic, or missense small nucleotide polymorphisms (SNPs) have been reported for the *hP2RX7* [133]. Macrophages/microglia of various species, including humans, are endowed with gain-of-function or loss-of-function P2X7R SNPs, with important consequences for their physiology/pathophysiology.

Early investigations characterized various polymorphisms, but did not couple their identification with population genetic studies in order to define possible illnesses linked to these mutations. The rs3751143 polymorphic mutant of *P2RX7* coding for the E496A-P2X7R impaired the ATP-induced IL-1β release from human monocytes [134]. It was shown for this SNP that monocytes expressed a non-functional receptor; when transfected into HEK293 cells, at low density, the receptor was non-functional, but regained function at a high receptor density [135]. Apparently, the glutamic acid at position 496 was required for optimal assembly of the P2X7R. Another study characterized the gain-of-function SNP rs2297595 (A166G) and demonstrated that the cysteine-rich domain 1 of P2X7Rs is critical for regulating P2X7R pore function [133]. rs1718119 (A348T-P2X7R) combined with the wild-type P2X7R exhibited enhanced ATP-induced ethidium uptake [136]. The SNP rs28360457 (R307Q) owing a mutation within the ATP binding site caused a massive loss-of-function, especially when it was expressed together with rs1653624 (I568N) [137].

More recently, some of these SNPs were suggested to be involved in various illnesses. Especially, the killing of intracellular bacteria surviving in phagosomes within macrophages depended on undisturbed P2X7R function. It was found that the 1513C allele (coding for E486A; rs200141401) is a risk factor in the development of extrapulmonary and pulmonary tuberculosis in many ethnic populations [18,138,139]. Observation of low P2X7R function in subjects with symptomatic *Toxoplasma gondii*-infected human macrophages showed that the loss-of-function rs3751143 SNP (E496A) significantly reduced P2X7R-mediated parasite killing [140,141].

The risk of age-related macular degeneration was increased by the existence of a rare functional haplotype of the *P2RX4* and *P2RX7* genes in macrophages [142]. Whereas the transfection of wild-type P2X7R in HEK293 cells conferred robust phagocytosis towards latex beads, co-expression of T315C-P2X7R with the G150R-P2X7R (rs28360447) almost completely inhibited phagocytotic activity. Thus, the impairment of the normal scavenger function of macrophages and microglia impaired removal of subretinal deposits and predisposed individuals towards macular degeneration.

A particularly interesting issue is the role of *P2RX7* polymorphism in osteoclast pathophysiology [143]. Osteoclasts are bone cells that are derived from the hematopoietic lineage and are functionally/biologically closely related to macrophages [144]. Genetic linkage studies have shown a clear association between *P2RX7* SNPs in the development of bone loss, osteoporosis, and risk of fractures [145]. Bone mass reduction was found to be, e.g., associated with the loss-of-function SNPs rs1718119 (A348T) and rs3751143 (E496A) [146].

Linkage studies also suggested that SNPs of *P2RX7* are associated with diverse psychiatric and neurological illnesses. In this case, a microglia-based neuro-inflammatory reaction might be causally involved, e.g., in mood disorders. It has been suggested that the SNP rs2230912 coding for the Q460R-P2X7R indicates a predisposition for major depressive disorder (MDD) [66,147,148]. Nonetheless, in the meantime, numerous clinical data failed to confirm this assumption [149,150]. In accordance with the doubts cast on the causal relationship between Q460R-P2X7R and MDD, the ATP-induced inward current was the same through the wild-type P2X7R and the Q460R polymorphic receptor when transfected into HEK293 cells [151]. However, co-expression of the wild-type receptor with the Q460R polymorphic receptor resulted in inhibition of calcium influx and current response to ATP [152]. Similarly, conditional humanized mice co-expressing both P2X7R variants showed alterations in their sleep quality resembling signs of a prodromal MDD state [153]. In conclusion, haplotypes formed between various *P2RX7* SNPs rather than a single receptor-polymorphism was thought to be responsible for MDD predisposition [154].

MDD and bipolar disorder are aggregated in families, and epidemiological studies have found in both cases evidence for genetic susceptibility. However, in the case of bipolar disorder a different *P2RX7* polymorphism (rs1718119 coding for A348T) was reported to be involved in this disease in contrast to that responsible for MDD [148,155].

With respect to neurological illnesses the rare SNP rs28360457 (R307Q) with absent pore formation was suggested to protect against neuroinflammation in multiple sclerosis [156]. The R307Q-P2X7R responsible for this effect was found to be located at monocytes/macrophages.

## 8. Conclusions

P2X7Rs are highly expressed on macrophages, appear to be crucial for proliferation, differentiation, and apoptosis of this cell type, and are involved in the macrophage immune response to pathogens and peripheral/central disorders of diverse origin. The activation of P2X7Rs initiates pore formation in the plasma membrane of macrophages allowing the entry of large cations into the intracellular space; this leads through interaction with pannexin-1, TRPA1, P2X4, and ANO6 Cl^−^ channels to inflammasome activation, and consequently, (neuro)inflammation (Figure 3). The assembly of the P2X7R-NLRP3-caspase-1 complex plays a key role in the induction/progression of rheumatoid arthritis, Crohn’s disease, liver fibrosis, sepsis, renal inflammation, pulmonary inflammation, and the amplification of neurodegenerative or mood diseases. In conclusion, P2X7Rs might be therapeutic targets to preserve immune homeostasis via their function on macrophages.

## Data Availability

Not applicable.

## References

[1] Burnstock G. (1972). Purinergic nerves. Pharmacol. Rev..

[2] Huang Z., Xie N., Illes P., Di Virgilio F., Ulrich H., Semyanov A., Verkhratsky A., Sperlagh B., Yu S.-G., Huang C. (2021). From purines to purinergic signalling: Molecular functions and human diseases. Signal Transduct. Target. Ther..

[3] Abbracchio M.P., Burnstock G. (1994). Purinoceptors: Are there families of P2X and P2Y purinoceptors?. Pharmacol. Ther..

[4] Burnstock G. (2017). Purinergic Signaling in the Cardiovascular System. Circ. Res..

[5] Jarvis M.F., Khakh B.S. (2009). ATP-gated P2X cation-channels. Neuropharmacology.

[6] Abbracchio M.P., Burnstock G., Boeynaems J.M., Barnard E.A., Boyer J.L., Kennedy C., Knight G.E., Fumagalli M., Gachet C., Jacobson K.A. (2006). International Union of Pharmacology, L.V.III: Update on the P2Y G protein-coupled nucleotide receptors: From molecular mechanisms and pathophysiology to therapy. Pharmacol. Rev..

[7] Jacobson K.A., Delicado E.G., Gachet C., Kennedy C., von Kügelgen I., Li B., Miras-Portugal M.T., Novak I., Schöneberg T., Perez-Sen R. (2020). Update of P2Y receptor pharmacology: IUPHAR Review 27. Br. J. Pharmacol..

[8] Fredholm B.B., IJzerman A.P., Jacobson K.A., Linden J., Müller C.E. (2011). International Union of Basic and Clinical Pharmacology. LXXXI. Nomenclature and classification of adenosine receptors—An update. Pharmacol. Rev..

[9] Zimmermann H., Zebisch M., Sträter N. (2012). Cellular function and molecular structure of ecto-nucleotidases. Purinergic Signal..

[10] Yegutkin G.G. (2014). Enzymes involved in metabolism of extracellular nucleotides and nucleosides: Functional implications and measurement of activities. Crit. Rev. Biochem. Mol. Biol..

[11] Zimmermann H., Braun N. (1999). Ecto-nucleotidases--molecular structures, catalytic properties, and functional roles in the nervous system. Prog. Brain Res..

[12] Burnstock G., Boeynaems J.M. (2014). Purinergic signalling and immune cells. Purinergic Signal..

[13] Surprenant A., Rassendren F., Kawashima E., North R.A., Buell G. (1996). The cytolytic P2Z receptor for extracellular ATP identified as a P2X receptor (P2X7). Science.

[14] North R.A. (2002). Molecular physiology of P2X receptors. Physiol. Rev..

[15] Di Virgilio F., Dal Ben D., Sarti A.C., Giuliani A.L., Falzoni S. (2017). The P2X7 Receptor in Infection and Inflammation. Immunity.

[16] Di Virgilio F., Schmalzing G., Markwardt F. (2018). The Elusive P2X7 Macropore. Trends Cell Biol..

[17] Illes P., Rubini P., Ulrich H., Zhao Y., Tang Y. (2020). Regulation of Microglial Functions by Purinergic Mechanisms in the Healthy and Diseased CNS. Cells.

[18] Miller C.M., Boulter N.R., Fuller S.J., Zakrzewski A.M., Lees M.P., Saunders B.M., Wiley J.S., Smith N.C. (2011). The role of the P2X7 receptor in infectious diseases. PLoS Pathog..

[19] Lavin Y., Merad M. (2013). Macrophages: Gatekeepers of tissue integrity. Cancer Immunol. Res..

[20] Mosser D.M., Edwards J.P. (2008). Exploring the full spectrum of macrophage activation. Nat. Rev. Immunol..

[21] Jakubzick C.V., Randolph G.J., Henson P.M. (2017). Monocyte differentiation and antigen-presenting functions. Nat. Rev. Immunol..

[22] Wynn T.A., Chawla A., Pollard J.W. (2013). Macrophage biology in development, homeostasis and disease. Nature.

[23] Ginhoux F., Jung S. (2014). Monocytes and macrophages: Developmental pathways and tissue homeostasis. Nat. Rev. Immunol..

[24] Layhadi J.A., Fountain S.J. (2017). P2X4 Receptor-Dependent Ca^2+^ Influx in Model Human Monocytes and Macrophages. Int. J. Mol. Sci..

[25] Sica A., Mantovani A. (2012). Macrophage plasticity and polarization: In vivo veritas. J. Clin. Investig..

[26] Amici S.A., Dong J., Guerau-de-Arellano M. (2017). Molecular Mechanisms Modulating the Phenotype of Macrophages and Microglia. Front. Immunol..

[27] Kigerl K.A., Gensel J.C., Ankeny D.P., Alexander J.K., Donnelly D.J., Popovich P.G. (2009). Identification of two distinct macrophage subsets with divergent effects causing either neurotoxicity or regeneration in the injured mouse spinal cord. J. Neurosci..

[28] Klaver D., Thurnher M. (2021). Control of Macrophage Inflammation by P2Y Purinergic Receptors. Cells.

[29] Duluc D., Delneste Y., Tan F., Moles M.P., Grimaud L., Lenoir J., Preisser L., Anegon I., Catala L., Ifrah N. (2007). Tumor-associated leukemia inhibitory factor and IL-6 skew monocyte differentiation into tumor-associated macrophage-like cells. Blood.

[30] Martinez F.O., Sica A., Mantovani A., Locati M. (2008). Macrophage activation and polarization. Front. Biosci..

[31] Illes P., Müller C.E., Jacobson K.A., Grutter T., Nicke A., Fountain S.J., Kennedy C., Schmalzing G., Jarvis M.F., Stojilkovic S.S. (2021). Update of P2X receptor properties and their pharmacology: IUPHAR Review 30. Br. J. Pharmacol..

[32] Buell G., Chessell I.P., Michel A.D., Collo G., Salazzo M., Herren S., Gretener D., Grahames C., Kaur R., Kosco-Vilbois M.H. (1998). Blockade of human P2X7 receptor function with a monoclonal antibody. Blood.

[33] Burnstock G., Knight G.E. (2018). The potential of P2X7 receptors as a therapeutic target, including inflammation and tumour progression. Purinergic Signal..

[34] Mansoor S.E., Lü W., Oosterheert W., Shekhar M., Tajkhorshid E., Gouaux E. (2016). X-ray structures define human P2X3 receptor gating cycle and antagonist action. Nature.

[35] Hattori M., Gouaux E. (2012). Molecular mechanism of ATP binding and ion channel activation in P2X receptors. Nature.

[36] McCarthy A.E., Yoshioka C., Mansoor S.E. (2019). Full-Length P2X7 Structures Reveal How Palmitoylation Prevents Channel Desensitization. Cell.

[37] Kasuya G., Fujiwara Y., Takemoto M., Dohmae N., Nakada-Nakura Y., Ishitani R., Hattori M., Nureki O. (2016). Structural Insights into Divalent Cation Modulations of ATP-Gated P2X Receptor Channels. Cell Rep..

[38] Gudipaty L., Humphreys B.D., Buell G., Dubyak G.R. (2001). Regulation of P2X(7) nucleotide receptor function in human monocytes by extracellular ions and receptor density. Am. J. Physiol. Cell Physiol..

[39] Falzoni S., Munerati M., Ferrari D., Spisani S., Moretti S., Di Virgilio F. (1995). The purinergic P2Z receptor of human macrophage cells. Characterization and possible physiological role. J. Clin. Investig..

[40] Gu B.J., Zhang W.Y., Bendall L.J., Chessell I.P., Buell G.N., Wiley J.S. (2000). Expression of P2X7 purinoceptors on human lymphocytes and monocytes: Evidence for nonfunctional P2X7 receptors. Am. J. Physiol. Cell Physiol..

[41] Monif M., Burnstock G., Williams D.A. (2010). Microglia: Proliferation and activation driven by the P2X7 receptor. Int. J. Biochem. Cell Biol..

[42] Qin J., Zhang X., Tan B., Zhang S., Yin C., Xue Q., Zhang Z., Ren H., Chen J., Liu M. (2020). Blocking P2X7-Mediated Macrophage Polarization Overcomes Treatment Resistance in Lung Cancer. Cancer Immunol. Res..

[43] Raneia E Silva P.A., de Lima D.S., Mesquita Luiz J.P., Camara N.O.S., Alves-Filho J.C.F., Pontillo A., Bortoluci K.R., Faquim-Mauro E.L. (2021). Inflammatory effect of Bothropstoxin-I from Bothrops jararacussu venom mediated by NLRP3 inflammasome involves ATP and P2X7 receptor. Clin. Sci..

[44] Zhou Y., Fei M., Zhang G., Liang W.C., Lin W., Wu Y., Piskol R., Ridgway J., McNamara E., Huang H. (2020). Blockade of the Phagocytic Receptor MerTK on Tumor-Associated Macrophages Enhances P2X7R-Dependent, STING Activation by Tumor-Derived cGAMP. Immunity.

[45] Gallenga C.E., Lonardi M., Pacetti S., Violanti S.S., Tassinari P., Di Virgilio F., Tognon M., Perri P. (2021). Molecular Mechanisms Related to Oxidative Stress in Retinitis Pigmentosa. Antioxidants.

[46] Xu S.L., Lin Y., Liu W., Zhu X.Z., Liu D., Tong M.L., Liu L.L., Lin L.R. (2020). The P2X7 receptor mediates NLRP3-dependent IL-1beta secretion and promotes phagocytosis in the macrophage response to Treponema pallidum. Int. Immunopharmacol..

[47] Janks L., Sprague R.S., Egan T.M. (2019). ATP-Gated P2X7 Receptors Require Chloride Channels To Promote Inflammation in Human Macrophages. J. Immunol..

[48] Hempel C., Nörenberg W., Sobottka H., Urban N., Nicke A., Fischer W., Schaefer M. (2013). The phenothiazine-class antipsychotic drugs prochlorperazine and trifluoperazine are potent allosteric modulators of the human P2X7 receptor. Neuropharmacology.

[49] Nurkhametova D., Siniavin A., Streltsova M., Kudryavtsev D., Kudryavtsev I., Giniatullina R., Tsetlin V., Malm T., Giniatullin R. (2020). Does Cholinergic Stimulation Affect the P2X7 Receptor-Mediated Dye Uptake in Mast Cells and Macrophages?. Front. Cell. Neurosci..

[50] Schachter J., Motta A.P., de Souza Z.A., da Silva-Souza H.A., Guimaraes M.Z., Persechini P.M. (2008). ATP-induced P2X7-associated uptake of large molecules involves distinct mechanisms for cations and anions in macrophages. J. Cell Sci..

[51] Yang C., Shi S., Su Y., Tong J.S., Li L. (2020). P2X7R promotes angiogenesis and tumour-associated macrophage recruitment by regulating the, N.F.-κB signalling pathway in colorectal cancer cells. J. Cell. Mol. Med..

[52] Zhang Y., Jiang M., Cui B.W., Jin C.H., Wu Y.L., Shang Y., Yang H.X., Wu M., Liu J., Qiao C.Y. (2020). P2X7 receptor-targeted regulation by tetrahydroxystilbene glucoside in alcoholic hepatosteatosis: A new strategy towards macrophage-hepatocyte crosstalk. Br. J. Pharmacol..

[53] Dong X., Zheng Z., Lin P., Fu X., Li F., Jiang J., Jiang J., Zhu P. (2020). ACPAs promote IL-1-beta production in rheumatoid arthritis by activating the NLRP3 inflammasome. Cell. Mol. Immunol..

[54] Ousingsawat J., Wanitchakool P., Kmit A., Romao A.M., Jantarajit W., Schreiber R., Kunzelmann K. (2015). Anoctamin 6 mediates effects essential for innate immunity downstream of P2X7 receptors in macrophages. Nat. Commun..

[55] Moreira-Souza A.C.A., Almeida-da-Silva C.L.C., Rangel T.P., Rocha G.D.C., Bellio M., Zamboni D.S., Vommaro R.C., Coutinho-Silva R. (2017). The P2X7 Receptor Mediates Toxoplasma gondii Control in Macrophages through Canonical NLRP3 Inflammasome Activation and Reactive Oxygen Species Production. Front. Immunol..

[56] Marques-da-Silva C., Chaves M.M., Castro N.G., Coutinho-Silva R., Guimaraes M.Z. (2011). Colchicine inhibits cationic dye uptake induced by ATP in P2X2 and P2X7 receptor-expressing cells: Implications for its therapeutic action. Br. J. Pharmacol..

[57] Janks L., Sharma C.V.R., Egan T.M. (2018). A central role for P2X7 receptors in human microglia. J. Neuroinflammation.

[58] Yaron J.R., Gangaraju S., Rao M.Y., Kong X., Zhang L., Su F., Tian Y., Glenn H.L., Meldrum D.R. (2015). K^+^ regulates Ca^2+^ to drive inflammasome signaling: Dynamic visualization of ion flux in live cells. Cell Death Dis..

[59] Pelegrin P., Surprenant A. (2006). Pannexin-1 mediates large pore formation and interleukin-1beta release by the ATP-gated P2X7 receptor. EMBO J..

[60] Pfeiffer Z.A., Aga M., Prabhu U., Watters J.J., Hall D.J., Bertics P.J. (2004). The nucleotide receptor P2X7 mediates actin reorganization and membrane blebbing in, R.A.W 264.7 macrophages via p38 MAP kinase and Rho. J. Leukoc. Biol..

[61] Noguchi T., Ishii K., Fukutomi H., Naguro I., Matsuzawa A., Takeda K., Ichijo H. (2008). Requirement of reactive oxygen species-dependent activation of ASK1-p38 MAPK pathway for extracellular ATP-induced apoptosis in macrophage. J. Biol. Chem..

[62] Le S.H., Raymond M.N. (2007). P2X7 receptor-mediated phosphatidic acid production delays ATP-induced pore opening and cytolysis of, R.A.W 264.7 macrophages. Cell. Signal..

[63] Thomas L.M., Salter R.D. (2010). Activation of macrophages by P2X7-induced microvesicles from myeloid cells is mediated by phospholipids and is partially dependent on, T.L.R4. J. Immunol..

[64] Matty M.A., Knudsen D.R., Walton E.M., Beerman R.W., Cronan M.R., Pyle C.J., Hernandez R.E., Tobin D.M. (2019). Potentiation of P2RX7 as a host-directed strategy for control of mycobacterial infection. Elife.

[65] Bomfim C.C.B., Amaral E.P., Cassado A.D.A., Salles A., do Nascimento R.S., Lasunskaia E., Hirata M.H., Álvarez J.M., D’Império-Lima M.R. (2017). P2X7 Receptor in Bone Marrow-Derived Cells Aggravates Tuberculosis Caused by Hypervirulent Mycobacterium bovis. Front. Immunol..

[66] Illes P., Verkhratsky A., Tang Y. (2019). Pathological ATPergic Signaling in Major Depression and Bipolar Disorder. Front. Mol. Neurosci..

[67] Virginio C., MacKenzie A., Rassendren F.A., North R.A., Surprenant A. (1999). Pore dilation of neuronal P2X receptor channels. Nat. Neurosci..

[68] Li M., Toombes G.E., Silberberg S.D., Swartz K.J. (2015). Physical basis of apparent pore dilation of ATP-activated P2X receptor channels. Nat. Neurosci..

[69] Pippel A., Stolz M., Woltersdorf R., Kless A., Schmalzing G., Markwardt F. (2017). Localization of the gate and selectivity filter of the full-length P2X7 receptor. Proc. Natl. Acad. Sci. USA.

[70] Markwardt F. (2021). Human P2X7 receptors-Properties of single ATP-gated ion channels. Biochem. Pharmacol..

[71] Kopp R., Krautloher A., Ramirez-Fernandez A., Nicke A. (2019). P2X7 Interactions and Signaling-Making Head or Tail of It. Front. Mol. Neurosci..

[72] Smart M.L., Gu B., Panchal R.G., Wiley J., Cromer B., Williams D.A., Petrou S. (2003). P2X7 receptor cell surface expression and cytolytic pore formation are regulated by a distal C-terminal region. J. Biol. Chem..

[73] Panchin Y., Kelmanson I., Matz M., Lukyanov K., Usman N., Lukyanov S. (2000). A ubiquitous family of putative gap junction molecules. Curr. Biol..

[74] Pelegrin P., Surprenant A. (2009). The P2X7 receptor-pannexin connection to dye uptake and IL-1beta release. Purinergic Signal..

[75] Alberto A.V., Faria R.X., Couto C.G., Ferreira L.G., Souza C.A., Teixeira P.C., Fróes M.M., Alves L.A. (2013). Is pannexin the pore associated with the P2X7 receptor?. Naunyn Schmiedebergs Arch. Pharmacol..

[76] Jung B.C., Kim S.H., Lim J., Kim Y.S. (2020). Activation of pannexin-1 mediates triglyceride-induced macrophage cell death. BMB Rep..

[77] Garre J.M., Yang G., Bukauskas F.F., Bennett M.V. (2016). FGF-1 Triggers Pannexin-1 Hemichannel Opening in Spinal Astrocytes of Rodents and Promotes Inflammatory Responses in Acute Spinal Cord Slices. J. Neurosci..

[78] Perez-Flores G., Lévesque S.A., Pacheco J., Vaca L., Lacroix S., Pérez-Cornejo P., Arreola J. (2015). The P2X7/P2X4 interaction shapes the purinergic response in murine macrophages. Biochem. Biophys. Res. Commun..

[79] Karasawa A., Michalski K., Mikhelzon P., Kawate T. (2017). The P2X7 receptor forms a dye-permeable pore independent of its intracellular domain but dependent on membrane lipid composition. Elife.

[80] Dunning K., Martz A., Peralta F.A., Cevoli F., Boue-Grabot E., Compan V., Gautherat F., Wolf P., Chataigneau T., Grutter T. (2021). P2X7 Receptors and, T.M.EM16 Channels Are Functionally Coupled with Implications for Macropore Formation and Current Facilitation. Int. J. Mol. Sci..

[81] Harkat M., Peverini L., Cerdan A.H., Dunning K., Beudez J., Martz A., Calimet N., Specht A., Cecchini M., Chataigneau T. (2017). On the permeation of large organic cations through the pore of, ATP-gated P2X receptors. Proc. Natl. Acad. Sci. USA.

[82] Babiychuk E.B., Monastyrskaya K., Potez S., Draeger A. (2011). Blebbing confers resistance against cell lysis. Cell Death Differ..

[83] Taylor S.R., Turner C.M., Elliott J.I., McDaid J., Hewitt R., Smith J., Pickering M.C., Whitehouse D.L., Cook H.T., Burnstock G. (2009). P2X7 deficiency attenuates renal injury in experimental glomerulonephritis. J. Am. Soc. Nephrol..

[84] Jiang L.H., Rassendren F., MacKenzie A., Zhang Y.H., Surprenant A., North R.A. (2005). N-methyl-D-glucamine and propidium dyes utilize different permeation pathways at rat P2X7 receptors. Am. J. Physiol. Cell Physiol..

[85] Cui X., Wan B., Yang Y., Ren X., Guo L.H., Zhang H. (2016). Crucial Role of P2X7 Receptor in Regulating Exocytosis of Single-Walled Carbon Nanotubes in Macrophages. Small.

[86] Rigato C., Swinnen N., Buckinx R., Couillin I., Mangin J.M., Rigo J.M., Legendre P., Le Corronc H. (2012). Microglia proliferation is controlled by P2X7 receptors in a Pannexin-1-independent manner during early embryonic spinal cord invasion. J. Neurosci..

[87] Monif M., Reid C.A., Powell K.L., Drummond K.J., O’Brien T.J., Williams D.A. (2016). Interleukin-1beta has trophic effects in microglia and its release is mediated by P2X7R pore. J. Neuroinflammation.

[88] Gheorghe R.O., Deftu A., Filippi A., Grosu A., Bica-Popi M., Chiritoiu M., Chiritoiu G., Munteanu C., Silvestro L., Ristoiu V. (2020). Silencing the Cytoskeleton Protein Iba1 (Ionized Calcium Binding Adapter Protein 1) Interferes with, B.V.2 Microglia Functioning. Cell. Mol. Neurobiol..

[89] Savio L.E.B., de Andrade M.P., da Silva C.G., Coutinho-Silva R. (2018). The P2X7 Receptor in Inflammatory Diseases: Angel or Demon?. Front. Pharmacol..

[90] Shao B.Z., Xu Z.Q., Han B.Z., Su D.F., Liu C. (2015). NLRP3 inflammasome and its inhibitors: A review. Front Pharmacol..

[91] Young C.N.J., Gorecki D.C. (2018). P2RX7 Purinoceptor as a Therapeutic Target-The Second Coming?. Front. Chem..

[92] Perregaux D.G., Gabel C.A. (1998). Post-translational processing of murine IL-1: Evidence that ATP-induced release of IL-1 alpha and IL-1 beta occurs via a similar mechanism. J. Immunol..

[93] Abderrazak A., Syrovets T., Couchie D., El H.K., Friguet B., Simmet T., Rouis M. (2015). NLRP3 inflammasome: From a danger signal sensor to a regulatory node of oxidative stress and inflammatory diseases. Redox Biol..

[94] Jeong Y.H., Walsh M.C., Yu J., Shen H., Wherry E.J., Choi Y. (2020). Mice Lacking the Purinergic Receptor P2X5 Exhibit Defective Inflammasome Activation and Early Susceptibility to Listeria monocytogenes. J. Immunol..

[95] Li L.H., Chen T.L., Chiu H.W., Hsu C.H., Wang C.C., Tai T.T., Ju T.C., Chen F.H., Chernikov O.V., Tsai W.C. (2020). Critical Role for the NLRP3 Inflammasome in Mediating IL-1beta Production in Shigella sonnei-Infected Macrophages. Front. Immunol..

[96] Munoz-Planillo R., Kuffa P., Martinez-Colon G., Smith B.L., Rajendiran T.M., Nunez G. (2013). K^+^ efflux is the common trigger of NLRP3 inflammasome activation by bacterial toxins and particulate matter. Immunity.

[97] Brough D., Le Feuvre R.A., Wheeler R.D., Solovyova N., Hilfiker S., Rothwell N.J., Verkhratsky A. (2003). Ca^2+^ stores and Ca^2+^ entry differentially contribute to the release of IL-1 beta and IL-1 alpha from murine macrophages. J. Immunol..

[98] Kahlenberg J.M., Dubyak G.R. (2004). Mechanisms of caspase-1 activation by P2X7 receptor-mediated K+ release. Am. J. Physiol. Cell Physiol..

[99] Hanley P.J., Kronlage M., Kirschning C., Del R.A., Di Virgilio F., Leipziger J., Chessell I.P., Sargin S., Filippov M.A., Lindemann O. (2012). Transient P2X7 receptor activation triggers macrophage death independent of Toll-like receptors 2 and 4, caspase-1, and pannexin-1 proteins. J. Biol. Chem..

[100] Le Feuvre R.A., Brough D., Iwakura Y., Takeda K., Rothwell N.J. (2002). Priming of macrophages with lipopolysaccharide potentiates P2X7-mediated cell death via a caspase-1-dependent mechanism, independently of cytokine production. J. Biol. Chem..

[101] Csoka B., Németh Z.H., Törö G., Idzko M., Zech A., Koscsó B., Spolarics Z., Antonioli L., Cseri K., Erdélyi K. (2015). Extracellular ATP protects against sepsis through macrophage P2X7 purinergic receptors by enhancing intracellular bacterial killing. FASEB J..

[102] Wang W., Hu D., Feng Y., Wu C., Song Y., Liu W., Li A., Wang Y., Chen K., Tian M. (2020). Paxillin mediates ATP-induced activation of P2X7 receptor and NLRP3 inflammasome. BMC Biol..

[103] Tian C., Han X., He L., Tang F., Huang R., Lin Z., Li S., Deng S., Xu J., Huang H. (2020). Transient receptor potential ankyrin 1 contributes to the ATP-elicited oxidative stress and inflammation in THP-1-derived macrophage. Mol. Cell. Biochem..

[104] Wanke D., Mauch-Mücke K., Holler E., Hehlgans T. (2016). Human beta-defensin-2 and -3 enhance pro-inflammatory cytokine expression induced by, T.L.R ligands via ATP-release in a P2X7R dependent manner. Immunobiology.

[105] de Torre-Minguela C., Barbera-Cremades M., Gomez A.I., Martin-Sanchez F., Pelegrin P. (2016). Macrophage activation and polarization modify P2X7 receptor secretome influencing the inflammatory process. Sci. Rep..

[106] Pelegrin P., Surprenant A. (2009). Dynamics of macrophage polarization reveal new mechanism to inhibit IL-1beta release through pyrophosphates. EMBO J..

[107] Lopez-Castejon G., Baroja-Mazo A., Pelegrin P. (2011). Novel macrophage polarization model: From gene expression to identification of new anti-inflammatory molecules. Cell. Mol. Life Sci..

[108] Neves A.R., Castelo-Branco M.T., Figliuolo V.R., Bernardazzi C., Buongusto F., Yoshimoto A., Nanini H.F., Coutinho C.M., Carneiro A.J., Coutinho-Silva R. (2014). Overexpression of ATP-activated P2X7 receptors in the intestinal mucosa is implicated in the pathogenesis of Crohn’s disease. Inflamm. Bowel Dis..

[109] Jiang S., Zhang Y., Zheng J.H., Li X., Yao Y.L., Wu Y.L., Song S.Z., Sun P., Nan J.X., Lian L.H. (2017). Potentiation of hepatic stellate cell activation by extracellular ATP is dependent on P2X7R-mediated NLRP3 inflammasome activation. Pharmacol. Res..

[110] Jiang M., Cui B.W., Wu Y.L., Zhang Y., Shang Y., Liu J., Yang H.X., Qiao C.Y., Zhan Z.Y., Ye H. (2020). P2X7R orchestrates the progression of murine hepatic fibrosis by making a feedback loop from macrophage to hepatic stellate cells. Toxicol. Lett..

[111] Wu X., Ren J., Chen G., Wu L., Song X., Li G., Deng Y., Wang G., Gu G., Li J. (2017). Systemic blockade of P2X7 receptor protects against sepsis-induced intestinal barrier disruption. Sci. Rep..

[112] Pereira J.M.S., Barreira A.L., Gomes C.R., Ornellas F.M., Ornellas D.S., Miranda L.C., Cardoso L.R., Coutinho-Silva R., Schanaider A., Morales M.M. (2020). Brilliant blue, G.; a P2X7 receptor antagonist, attenuates early phase of renal inflammation, interstitial fibrosis and is associated with renal cell proliferation in ureteral obstruction in rats. BMC Nephrol..

[113] Koo T.Y., Lee J.G., Yan J.J., Jang J.Y., Ju K.D., Han M., Oh K.H., Ahn C., Yang J. (2017). The P2X7 receptor antagonist, oxidized adenosine triphosphate, ameliorates renal ischemia-reperfusion injury by expansion of regulatory T cells. Kidney Int..

[114] Kang M.J., Jo S.G., Kim D.J., Park J.H. (2017). NLRP3 inflammasome mediates interleukin-1beta production in immune cells in response to Acinetobacter baumannii and contributes to pulmonary inflammation in mice. Immunology.

[115] Ye Q., Wang B., Mao J. (2020). The pathogenesis and treatment of the ‘Cytokine Storm’ in, C.O.VID-19. J. Infect..

[116] Wang C., Xie J., Zhao L., Fei X., Zhang H., Tan Y., Nie X., Zhou L., Liu Z., Ren Y. (2020). Alveolar macrophage dysfunction and cytokine storm in the pathogenesis of two severe, C.O.VID-19 patients. EBioMedicine.

[117] Mehta P., McAuley D.F., Brown M., Sanchez E., Tattersall R.S., Manson J.J. (2020). COVID-19: Consider cytokine storm syndromes and immunosuppression. Lancet.

[118] Monteagudo L.A., Boothby A., Gertner E. (2020). Continuous Intravenous Anakinra Infusion to Calm the Cytokine Storm in Macrophage Activation Syndrome. ACR Open Rheumatol..

[119] Di Virgilio F., Tang Y., Sarti A.C., Rossato M. (2020). A rationale for targeting the P2X7 receptor in Coronavirus disease 19. Br. J. Pharmacol..

[120] Illes P. (2020). P2X7 Receptors Amplify CNS Damage in Neurodegenerative Diseases. Int. J. Mol. Sci..

[121] Illes P., Rubini P., Huang L., Tang Y. (2019). The P2X7 receptor: A new therapeutic target in Alzheimer’s disease. Expert Opin. Ther. Targets.

[122] Francistiova L., Bianchi C., Di Lauro C., Sebastian-Serrano A., de Diego-Garcia L., Kobolak J., Dinnyés A., Díaz-Hernández M. (2020). The Role of P2X7 Receptor in Alzheimer’s Disease. Front. Mol. Neurosci..

[123] Wang X.H., Xie X., Luo X.G., Shang H., He Z.Y. (2017). Inhibiting purinergic P2X7 receptors with the antagonist brilliant blue G is neuroprotective in an intranigral lipopolysaccharide animal model of Parkinson’s disease. Mol. Med. Rep..

[124] Oliveira-Giacomelli A., Albino M., de Souza H.D.N., Correa-Velloso J., de Jesus Santos A.P., Baranova J., Ulrich H. (2019). P2Y6 and P2X7 Receptor Antagonism Exerts Neuroprotective/ Neuroregenerative Effects in an Animal Model of Parkinson’s Disease. Front. Cell. Neurosci..

[125] Apolloni S., Amadio S., Parisi C., Matteucci A., Potenza R.L., Armida M., Popoli P., D’Ambrosi N., Volonté C. (2014). Spinal cord pathology is ameliorated by P2X7 antagonism in a, S.O.D1-mutant mouse model of amyotrophic lateral sclerosis. Dis. Models Mech..

[126] Fabbrizio P., Amadio S., Apolloni S., Volonte C. (2017). P2X7 Receptor Activation Modulates Autophagy in, S.O.D1-G93A Mouse Microglia. Front. Cell. Neurosci..

[127] Beaino W., Janssen B., Kooij G., van der Pol S.M.A., van Het H.B., van Horssen J., Windhorst A.D., de Vries H.E. (2017). Purinergic receptors P2Y12R and P2X7R: Potential targets for, P.E.T imaging of microglia phenotypes in multiple sclerosis. J. Neuroinflammation.

[128] Grygorowicz T., Struzynska L. (2019). Early P2X7R-dependent activation of microglia during the asymptomatic phase of autoimmune encephalomyelitis. Inflammopharmacology.

[129] Jimenez-Pacheco A., Diaz-Hernandez M., Arribas-Blazquez M., Sanz-Rodriguez A., Olivos-Ore L.A., Artalejo A.R., Alves M., Letavic M., Miras-Portugal M.T., Conroy R.M. (2016). Transient P2X7 Receptor Antagonism Produces Lasting Reductions in Spontaneous Seizures and Gliosis in Experimental Temporal Lobe Epilepsy. J. Neurosci..

[130] Beamer E., Fischer W., Engel T. (2017). The ATP-Gated P2X7 Receptor As a Target for the Treatment of Drug-Resistant Epilepsy. Front. Neurosci..

[131] Melani A., Amadio S., Gianfriddo M., Vannucchi M.G., Volonte C., Bernardi G., Pedata F., Sancesario G. (2006). P2X7 receptor modulation on microglial cells and reduction of brain infarct caused by middle cerebral artery occlusion in rat. J. Cereb. Blood Flow Metab..

[132] Bartlett R., Stokes L., Sluyter R. (2014). The P2X7 receptor channel: Recent developments and the use of P2X7 antagonists in models of disease. Pharmacol. Rev..

[133] Sun C., Chu J., Singh S., Salter R.D. (2010). Identification and characterization of a novel variant of the human P2X7 receptor resulting in gain of function. Purinergic Signal..

[134] Sluyter R., Shemon A.N., Wiley J.S. (2004). Glu496 to Ala polymorphism in the P2X7 receptor impairs, ATP-induced IL-1 beta release from human monocytes. J. Immunol..

[135] Gu B.J., Zhang W., Worthington R.A., Sluyter R., Dao-Ung P., Petrou S., Barden J.A., Wiley J.S. (2001). A Glu-496 to Ala polymorphism leads to loss of function of the human P2X7 receptor. J. Biol. Chem..

[136] Stokes L., Fuller S.J., Sluyter R., Skarratt K.K., Gu B.J., Wiley J.S. (2010). Two haplotypes of the P2X7 receptor containing the Ala-348 to Thr polymorphism exhibit a gain-of-function effect and enhanced interleukin-1beta secretion. FASEB J..

[137] Gu B.J., Sluyter R., Skarratt K.K., Shemon A.N., Dao-Ung L.P., Fuller S.J., Barden J.A., Clarke A.L., Petrou S., Wiley J.S. (2004). An Arg307 to Gln polymorphism within the ATP-binding site causes loss of function of the human P2X7 receptor. J. Biol. Chem..

[138] Xiao J., Sun L., Yan H., Jiao W., Miao Q., Feng W., Wu X., Gu Y., Jiao A., Guo Y. (2010). Metaanalysis of P2X7 gene polymorphisms and tuberculosis susceptibility. FEMS Immunol. Med. Microbiol..

[139] Britton W.J., Fernando S.L., Saunders B.M., Sluyter R., Wiley J.S. (2007). The genetic control of susceptibility to Mycobacterium tuberculosis. Novartis Found. Symp..

[140] Lees M.P., Fuller S.J., McLeod R., Boulter N.R., Miller C.M., Zakrzewski A.M., Mui E.J., Witola W.H., Coyne J.J., Hargrave A.C. (2010). P2X7 receptor-mediated killing of an intracellular parasite, Toxoplasma gondii, by human and murine macrophages. J. Immunol..

[141] Wiley J.S., Sluyter R., Gu B.J., Stokes L., Fuller S.J. (2011). The human P2X7 receptor and its role in innate immunity. Tissue Antigens.

[142] Gu B.J., Baird P.N., Vessey K.A., Skarratt K.K., Fletcher E.L., Fuller S.J., Richardson A.J., Guymer R.H., Wiley J.S. (2013). A rare functional haplotype of the P2RX4 and P2RX7 genes leads to loss of innate phagocytosis and confers increased risk of age-related macular degeneration. FASEB J..

[143] Jorgensen N.R. (2019). Role of the purinergic P2X receptors in osteoclast pathophysiology. Curr. Opin. Pharmacol..

[144] Boyle W.J., Simonet W.S., Lacey D.L. (2003). Osteoclast differentiation and activation. Nature.

[145] Jorgensen N.R., Syberg S., Ellegaard M. (2015). The role of P2X receptors in bone biology. Curr. Med. Chem..

[146] Varley I., Hughes D.C., Greeves J.P., Fraser W.D., Sale C. (2018). SNPs in the vicinity of P2X7R, RANK/RANKL/OPG and Wnt signalling pathways and their association with bone phenotypes in academy footballers. Bone.

[147] McQuillin A., Bass N.J., Choudhury K., Puri V., Kosmin M., Lawrence J., Curtis D., Gurling H.M. (2009). Case-control studies show that a non-conservative amino-acid change from a glutamine to arginine in the P2RX7 purinergic receptor protein is associated with both bipolar- and unipolar-affective disorders. Mol. Psychiatry.

[148] Czamara D., Müller-Myhsok B., Lucae S. (2018). The P2RX7 polymorphism rs2230912 is associated with depression: A meta-analysis. Prog. Neuropsychopharmacol. Biol. Psychiatry.

[149] Green E.K., Grozeva D., Raybould R., Elvidge G., Macgregor S., Craig I., Farmer A., McGuffin P., Forty L., Jones L. (2009). P2RX7: A bipolar and unipolar disorder candidate susceptibility gene?. Am. J. Med. Genet. B Neuropsychiatr. Genet..

[150] Feng W.P., Zhang B., Li W., Liu J. (2014). Lack of association of P2RX7 gene rs2230912 polymorphism with mood disorders: A meta-analysis. PLoS ONE.

[151] Roger S., Mei Z.Z., Baldwin J.M., Dong L., Bradley H., Baldwin S.A., Surprenant A., Jiang L.H. (2010). Single nucleotide polymorphisms that were identified in affective mood disorders affect ATP-activated P2X7 receptor functions. J. Psychiatr. Res..

[152] Aprile-Garcia F., Metzger M.W., Paez-Pereda M., Stadler H., Acuna M., Liberman A.C., Senin S.A., Gerez J., Hoijman E., Refojo D. (2016). Co-Expression of Wild-Type P2X7R with Gln460Arg Variant Alters Receptor Function. PLoS ONE.

[153] Metzger M.W., Walser S.M., Dedic N., Aprile-Garcia F., Jakubcakova V., Adamczyk M., Webb K.J., Uhr M., Refojo D., Schmidt M.V. (2017). Heterozygosity for the Mood Disorder-Associated Variant Gln460Arg Alters P2X7 Receptor Function and Sleep Quality. J. Neurosci..

[154] Sluyter R., Stokes L., Fuller S.J., Skarratt K.K., Gu B.J., Wiley J.S. (2010). Functional significance of P2RX7 polymorphisms associated with affective mood disorders. J. Psychiatr. Res..

[155] Backlund L., Nikamo P., Hukic D.S., Ek I.R., Träskman-Bendz L., Landén M., Edman G., Schalling M., Frisén L., Osby U. (2011). Cognitive manic symptoms associated with the P2RX7 gene in bipolar disorder. Bipolar Disord..

[156] Gu B.J., Field J., Dutertre S., Ou A., Kilpatrick T.J., Lechner-Scott J., Scott R., Lea R., Taylor B.V., Stankovich J. (2015). A rare P2X7 variant Arg307Gln with absent pore formation function protects against neuroinflammation in multiple sclerosis. Hum. Mol. Genet..

